# Cerebral metabolic derangements following traumatic brain injury

**DOI:** 10.1097/ACO.0000000000001183

**Published:** 2022-08-18

**Authors:** Simon Demers-Marcil, Jonathan P. Coles

**Affiliations:** a Division of Anaesthesia, University of Cambridge, Addenbrooke's Hospital, Cambridge, UK; b Department of Anesthesiology and Critical Care, CHU de Québec-Université Laval, Quebec City, Quebec, Canada

**Keywords:** cerebral metabolism, ischemia, microdialysis, microvascular ischemia, mitochondrial dysfunction, PET

## Abstract

Outcome following traumatic brain injury (TBI) remains variable, and derangements in cerebral metabolism are a common finding in patients with poor outcome. This review compares our understanding of cerebral metabolism in health with derangements seen following TBI.

**Recent findings:**

Ischemia is common within the first 24 h of injury and inconsistently detected by bedside monitoring. Metabolic derangements can also result from tissue hypoxia in the absence of ischemic reductions in blood flow due to microvascular ischemia and mitochondrial dysfunction. Glucose delivery across the injured brain is dependent on blood glucose and regional cerebral blood flow, and is an important contributor to derangements in glucose metabolism. Alternative energy substrates such as lactate, ketone bodies and succinate that may support mitochondrial function, and can be utilized when glucose availability is low, have been studied following TBI but require further investigation.

**Summary:**

Mitochondrial dysfunction and the use of alternative energy substrates are potential therapeutic targets, but improved understanding of the causes, impact and significance of metabolic derangements in clinical TBI are needed. Maintaining adequate oxygen and glucose delivery across the injured brain may accelerate the recovery of mitochondrial function and cerebral energy metabolism and remain important management targets.

## INTRODUCTION

Traumatic brain injury (TBI) still causes substantial suffering for individuals and their families with enormous costs for society. Those who require admission to intensive care with moderate and/or severe TBI based on a Glasgow Coma Score of 9–12 and less than 8, respectively, have the highest risk of death (20–30%), with 25% of survivors living with severe disability [1]. While outcome following TBI has improved through the delivery of protocolized management which aims to control intracranial pressure (ICP) and maintain cerebral oxygen delivery [2], it remains variable. In fact, bedside clinical monitoring of brain tissue oxygen and cerebral metabolism using microdialysis demonstrate evidence of persistent metabolic derangements which correlate with patient outcome [3^▪▪^]. Imaging studies using ^15^oxygen PET demonstrate reductions in cerebral blood flow (CBF) and increases in oxygen extraction fraction (OEF) which are consistent with ischemia, but this finding is less common beyond the first 24 h after injury [4^▪▪^,^5^^▪▪^]. At these later time points following TBI, metabolic derangements are more consistent with disruption of the microcirculation resulting in tissue hypoxia or low brain glucose delivery, and/or mitochondrial dysfunction [5^▪▪^]. In this review, we will examine how recent studies of oxygen and glucose delivery following TBI contribute to our understanding of derangements in cerebral energy metabolism in comparison with that seen within the normal brain. In addition, we will review recent studies that focus on how such data could help refine or develop future neuroprotective strategies that aim to improve functional outcome for patients with TBI. 

**Box 1:**
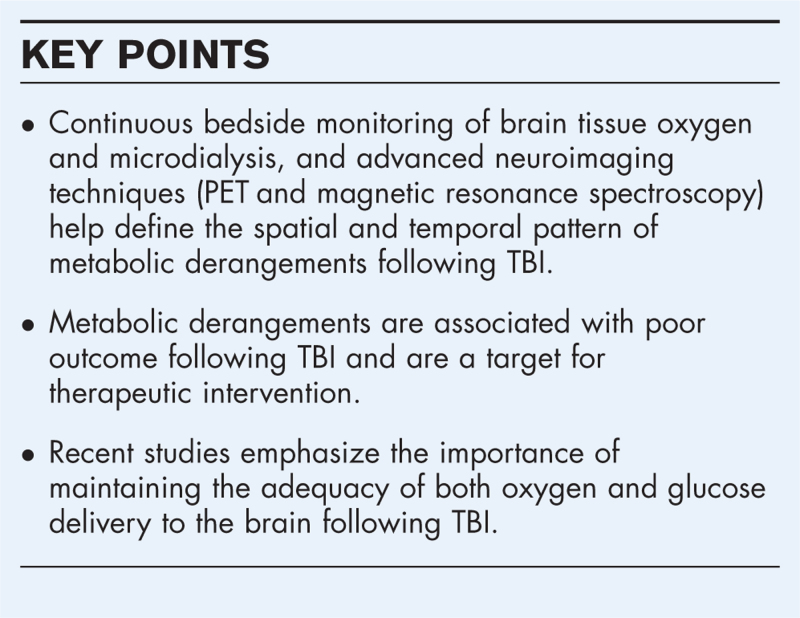
no caption available

## REFINEMENTS IN OUR UNDERSTANDING OF CEREBRAL METABOLISM IN HEALTH

While glycogen is produced and stored in astrocytes, the brain has limited reserves of metabolic substrate and requires a steady supply of both oxygen and glucose to support its needs. This dictates that regional increases in the cerebral metabolic rate of oxygen (CMRO_2_) and glucose (CMRG) must be matched by increases in CBF. This tight physiological relationship between supply and demand within the brain is termed flow-metabolism coupling. Blood glucose levels are tightly controlled and uptake across the blood–brain barrier is facilitated through the action of the GLUT1 transporter, resulting in brain tissue glucose levels which are approximately 20–50% of that found in arterial plasma [6]. Once within the cytosol, glucose is phosphorylated via hexokinase generating glucose-6-phosphate, trapping it within the cell and maintaining the concentration gradient for glucose uptake from the blood. The process through which glucose is subsequently metabolized is variable depending on the cell type concerned and the presence of sufficient oxygen. Glycolysis generates pyruvate and two molecules of ATP from glucose-6-phosphate, and under aerobic conditions pyruvate enters mitochondria where it is oxidized to CO_2_ and water. In the absence of sufficient oxygen, mitochondrial respiration is suspended and pyruvate is reduced to lactate which may remain within the cell to be metabolized later, or be released into the bloodstream. Indeed, large increases in the concentration of lactate in excess of pyruvate are a marker of anaerobic metabolism and are typically seen under conditions of tissue hypoxia and/or ischemia [7]. Glucose is also metabolized via the pentose-phosphate pathway (PPP) which is responsible for nucleic acid synthesis and has important antioxidant properties [8].

More recent studies demonstrate that neurons favor oxidative metabolism whereas glial cells (astrocytes and oligodendrocytes) produce lactate via glycolysis. The difference relates to the predominance of different forms of lactate dehydrogenase (LDH) found within the distinct cell types, with astrocytes producing pyruvate which is converted to lactate via LDH5 despite adequate oxygen tension [9]. These differences explain functional activation studies where increases in the uptake of glucose is found in astrocytes despite the fact that the majority of all energy expended within the brain occurs within neurons [9]. Current understanding is that glucose uptake and conversion to lactate within astrocytes is upregulated by a variety of mediators that include glutamate, and that the lactate generated is shuttled to neurons where it is metabolized aerobically. This is the basis of the astrocyte–neuron lactate transport shuttle where lactate is the predominant substrate utilized in the brain, and under normal conditions the lactate/pyruvate ratio (LPR) is typically greater than 10. Within neurons, lactate is converted to pyruvate which can be fully oxidized generating up to 36 ATP molecules within a process which generates reduced coenzymes [NADPH and flavin adenine dinucleotide (FADH_2_)]. These are oxidized by the transfer of electrons within the electron transport chain in mitochondria [10]. Finally, there is increasing recognition of the importance and role of lactate within the brain, not only as a substrate for energy metabolism, but as a chemical messenger resulting in changes in neuronal excitability that impact on a variety of physiological processes such as learning and memory [11].

Glucose, metabolized to lactate and pyruvate, is the predominant substrate for energy metabolism and the brain has very limited ability to use beta-oxidation of fatty acids to generate acetyl-CoA [12]. However, acetyl-CoA can be produced from ketone bodies, a product of fatty acid degradation and utilized in the brain as a substrate for ATP production. In the healthy human brain, the use of ketone bodies is mainly seen in a fasting state. Alternative substrates for cerebral energy production under conditions of stress are limited, but include limited stores of astrocytic glycogen [13] and glutamate [14].

## MONITORING CEREBRAL METABOLISM FOLLOWING TRAUMATIC BRAIN INJURY

**FIGURE 1:**
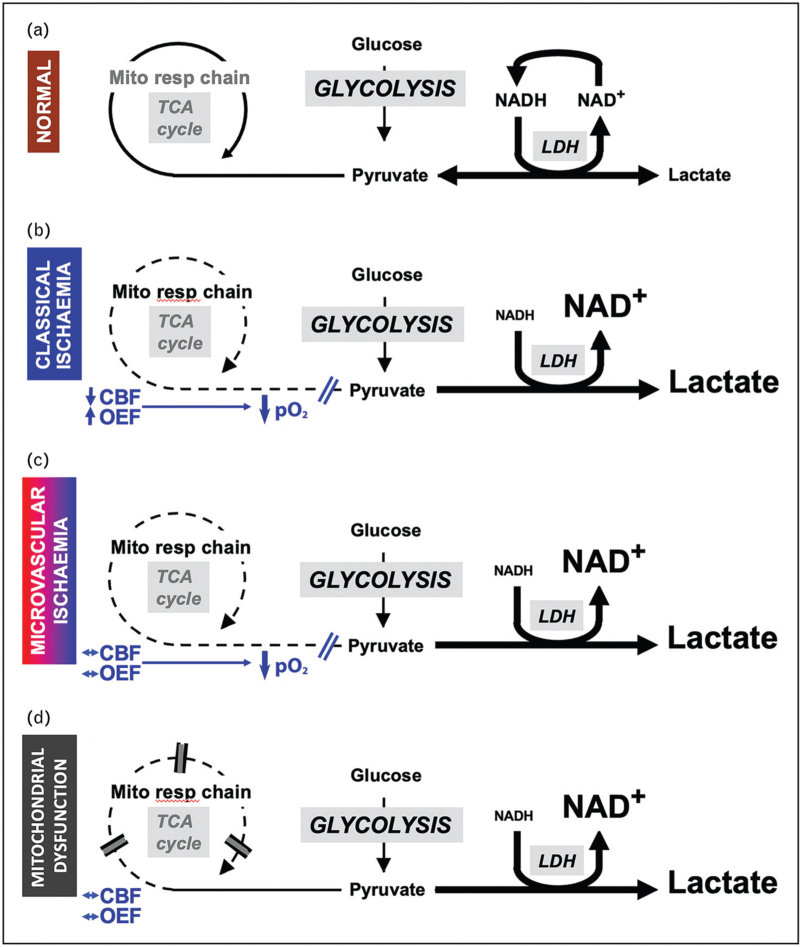
Schematic to demonstrate derangements in glucose metabolism following traumatic brain injury. Cerebral blood flow (CBF), lactate dehydrogenase (LDH), mitochondrial respiratory chain (Mito resp chain), nicotinamide adenine dinucleotide and its reduced form (NAD and NADH), oxygen extraction fraction (OEF), partial pressure of oxygen (pO_2_), TCA (tricarboxylic acid cycle). Under normal conditions both glucose and lactate can be metabolized to pyruvate which enters the tricarboxylic acid cycle and undergoes oxidative phosphorylation. While the concentration of lactate is higher than that of pyruvate the lactate/pyruvate ratio remains less than 20 (a). In classical ischemia the increase in oxygen extraction fraction that results from a reduction in cerebral blood flow can no longer maintain oxygen delivery to the brain and tissue pO_2_ falls preventing mitochondrial oxidative phosphorylation. Pyruvate is converted to lactate via lactate dehydrogenase generating NAD which is required to maintain increased glycolysis and energy production, and the lactate/pyruvate ratio increases to levels more than 25 (b). A similar increase in glycolysis resulting in a lactate/pyruvate ratio more than 25 occurs in microvascular ischemia where microvascular thrombosis, collapse and perivascular edema result in an increased diffusion barrier that limits oxygen delivery leading to low tissue pO_2_ (c), and mitochondrial dysfunction where oxidative phosphorylation is suspended despite maintenance of normal oxygen and glucose delivery (d). In both (c) and (d), average macrovascular cerebral blood flow and oxygen extraction fraction values are not typically ischemic. Modified with permission from that originally published in Menon DK, Ercole A. Critical care management of traumatic brain injury. Handb Clin Neurol 2017;140:239–74.

Within neurointensive care, continuous bedside multimodality monitoring includes devices placed within brain parenchyma via a cranial access device. These include probes providing measurement of ICP, tissue oxygen and cerebral metabolism which have helped refine our understanding of metabolic derangements following TBI [3^▪▪^,15^▪^]. Cerebral microdialysis samples brain extracellular fluid and can be analyzed at the bedside to provide hourly measurements of a variety of metabolites that include pyruvate, lactate, glucose, glycerol and glutamate. The LPR is the best studied metabolic marker in TBI and reflects the ratio of anaerobic to aerobic metabolism [16]. This measure can be used to define normal metabolism where the LPR is less than 20 [17], whereas an LPR more than 25 [16] is consistent with anaerobic metabolism (Fig. 1), and time spent above this threshold within the first 72 h post TBI has been shown to be associated with poor outcome [3^▪▪^]. Normal glucose microdialysate values in the brain are about 1.7 mmol/l [17], and studies show that both high and low brain glucose values are associated with worse outcome [3^▪▪^,^16^,^18^]. Brain tissue oxygen values (PbtO_2_) represent the balance between cerebral oxygen delivery and utilization, and a failure to maintain normoxia (∼18–22 mmHg) can result from systemic hypoxemia, inadequate perfusion and/or increased metabolic demand. Use of such focal monitoring tools has demonstrated episodes of tissue hypoxia and low brain glucose following clinical TBI, and the burden of such episodes are associated with poor outcome [2,3^▪▪^]. Recent data suggest a survival benefit in severe TBI patients when PbtO_2_ and ICP monitoring are combined as compared with ICP monitoring alone [2], and consensus guidelines for their use have recently been published [15^▪^].

Pathophysiological heterogeneity predicates the need for physiological imaging, and while both computed tomography and MRI can provide perfusion imaging, the gold standard technique for understanding cerebral metabolic derangements is PET. Use of multitracer oxygen-15 and ^18^F-fluorodeoxyglucose (^18^F-FDG) PET can provide whole brain imaging of CBF, blood volume, OEF, CMRO_2_ and CMRG, and calculation of kinetic parameters describing glucose delivery to the brain (K_1_) and glycolysis (k_3_). Noninvasive proton (^1^H-MRS) and phosphorus (^31^P-MRS) magnetic resonance spectroscopy can also be used to study cerebral metabolism. Such advanced imaging techniques can provide detailed understanding of the burden and spatial heterogeneity of metabolic derangements seen following TBI [4^▪▪^,^5^^▪▪^,^19^].

## DEFINING DERANGEMENTS IN CEREBRAL METABOLISM FOLLOWING TRAUMATIC BRAIN INJURY

**FIGURE 2:**
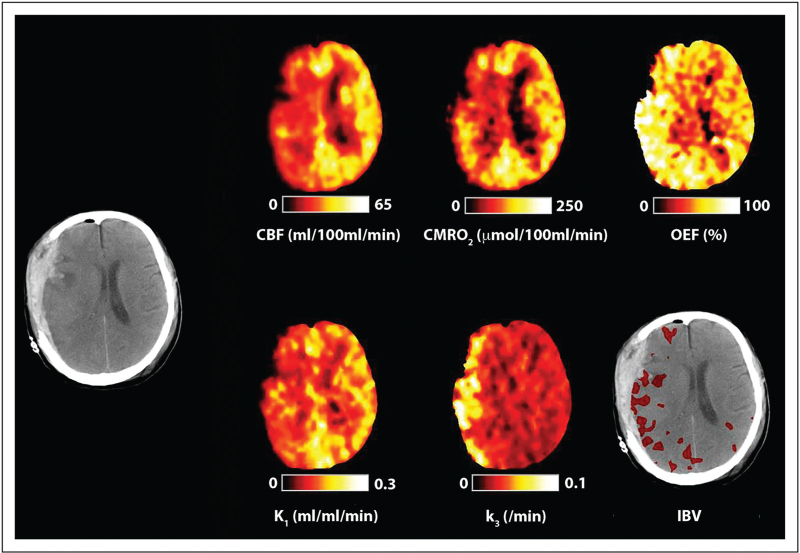
Cerebral ischemia. Computed tomography, cerebral blood flow, cerebral metabolic rate of oxygen, oxygen extraction fraction, ischemic brain volume (IBV) and PET ^18^F-fluorodeoxyglucose kinetic parameters for K_1_ (glucose delivery) and k_3_ (glycolysis) obtained in a 40-year-old female within 24 h of severe traumatic brain injury resulting from a fall. The computed tomography scan was obtained following evacuation of a subdural hematoma and demonstrates residual subdural blood with minimal midline shift and underlying hemorrhagic contusions. Cerebral blood flow, cerebral metabolic rate of oxygen and (K_1_) glucose delivery are reduced, whereas oxygen extraction fraction and k_3_ (glycolysis) are increased within the hemisphere underlying the subdural. These findings are consistent with classical ischemia.

Increases in the LPR associated with evidence of tissue hypoxia is indicative of ischemic glycolysis (Fig. 1b) and recent combined multitracer oxygen-15 and ^18^F-FDG PET studies confirm evidence of ischemic CBF reductions in acute TBI on the basis of a critical increase in both OEF and glycolysis (k_3_) (Fig. 2) [5^▪▪^]. The authors demonstrated that the ischemic brain volume (IBV) within the first 24 h following injury was associated with worse outcome using the Glasgow Outcome Score. While evidence of reversible ischemia is found within the vicinity of focal lesions and within brain that initially appears to be structurally normal for up to 10 days post injury, it was less common outside the first 24 h [4^▪▪^]. These data also highlight the extent of pathophysiological heterogeneity and confirm that bedside physiological monitoring of the adequacy of oxygen delivery should be interpreted with caution since global methods (jugular oximetry) may dilute and miss focal pathophysiology, whereas focal monitors (PbtO_2_) are critically dependent on sensor position.

Despite the evidence for classical macrovascular ischemia, brain tissue hypoxia can occur in the absence of CBF reductions and OEF increases consistent with ischemia. Menon *et al.*[20] previously demonstrated that brain regions with hypoxic tissue pO_2_ could have similar end capillary pO_2_ values to that found within normoxic regions, and could not increase oxygen extraction when challenged with a reduction in CBF. These findings were suggestive of an increased diffusion barrier preventing cellular oxygen delivery, and are consistent with evidence of perivascular edema, microvascular collapse and/or endothelial injury seen following TBI [20]. A more recent study used a PET ligand (^18^F-FMISO) which becomes covalently bound within hypoxic brain tissue to image tissue hypoxia across the whole of the injured brain [21]. This study confirmed that tissue hypoxia after TBI is not confined to regions with structural abnormality and can occur in the absence of conventional macrovascular ischemia. These findings describe a metabolic signature consistent with microvascular ischemia (Fig. 1c).

**FIGURE 3:**
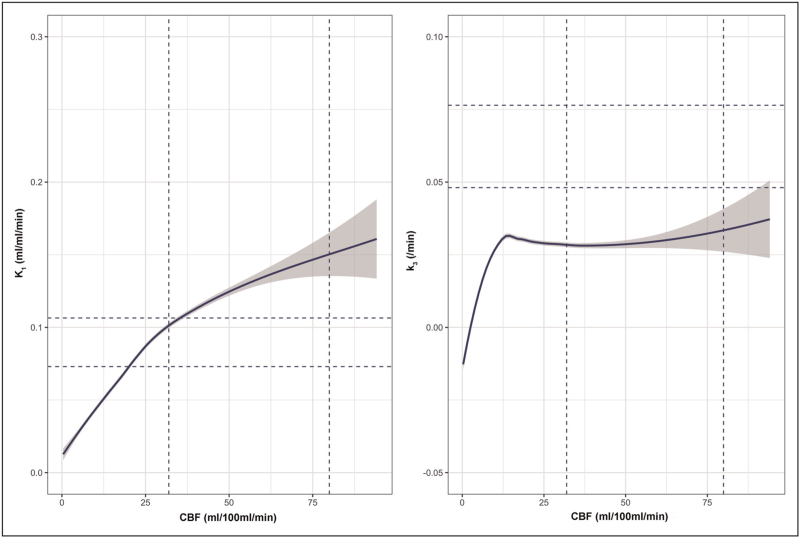
Glucose delivery and glycolysis are dependent on cerebral blood flow. The relationship between cerebral blood flow and ^18^F-fluorodeoxyglucose kinetic parameters for K_1_ (glucose delivery; left) and k_3_ (glycolysis; right) plotted for patients following traumatic brain injury. The fitted lines represent modeling of the relationship using locally weighted scatterplot smoothing, with the 95% confidence interval shown in gray. The vertical and horizontal dotted lines indicate the full range for healthy volunteer cerebral blood flow and ^18^F-fluorodeoxyglucose kinetic parameters, respectively.

While there is evidence of both macro and microvascular ischemia following TBI, PET studies typically demonstrate an overall reduction in cerebral glucose metabolism that is not explained by the effects of anesthesia, with regional increases in glycolysis associated with nonischemic CBF and CMRO_2_ reductions. The net result of these changes is that the metabolic ratio of oxygen-to-glucose utilization is reduced post TBI [5^▪▪^,^22^]. Nonischemic increases in glycolysis have also been found to be associated with low brain glucose delivery. Reductions in microdialysis glucose can be found despite normal plasma glucose providing evidence of impaired glucose delivery, which, like impaired oxygen delivery, may result from CBF reductions or microvascular dysfunction and/or cytotoxic edema [21]. Studies in TBI using ^18^F-FDG PET demonstrate that glucose delivery (K_1_) is generally maintained despite reductions in CBF, and is consistent with upregulation of GLUT1 transporters. Despite this, glucose delivery is clearly dependent on regional CBF, particularly as CBF falls less than 25 ml/100 ml/min, and glycolysis falls sharply with CBF less than 12 ml/100 ml/min (Fig. 3) [5^▪▪^,^22^]. Low plasma and microdialysis glucose may also reflect increased glucose demand as they are associated with increases in glycolysis within brain regions with nonischemic CBF reductions [5^▪▪^,^22^]. The accumulation of lactate under such circumstances may be beneficial as it can be metabolized in neurons as an energy substrate in the presence of oxygen [23], and in such cases the LPR would be expected to remain normal. A microdialysis study using perfusion of ^13^C-labeled glucose and metabolite analyses using high-resolution nuclear magnetic resonance spectroscopy (MRS) confirmed an increase in glycolysis, and that metabolism of glucose via the PPP tended to increase in association with tissue hypoxia signifying a switch to reparative processes following TBI [8]. Where microdialysis and advanced imaging (PET and ^31^P-MRS) show disturbed energy metabolism following TBI in the absence of ischemia, and despite an adequate supply of oxygen and glucose [24,25], it implies a metabolic signature that is consistent with mitochondrial dysfunction (Fig. 1d) [26].

## HOW CAN WE SUPPORT CEREBRAL ENERGY METABOLISM FOLLOWING TRAUMATIC BRAIN INJURY?

Derangements in cerebral metabolism can lead to lesion expansion and diffuse neuronal loss across the injured brain which may worsen functional outcome for patients. Preventing ischemia through optimizing ICP control and maintaining oxygen delivery are well recognized targets [4^▪▪^], and recent management guidelines stress the benefits of combining ICP, tissue oxygen and microdialysis guided management [16].

### Glucose delivery

**FIGURE 4:**
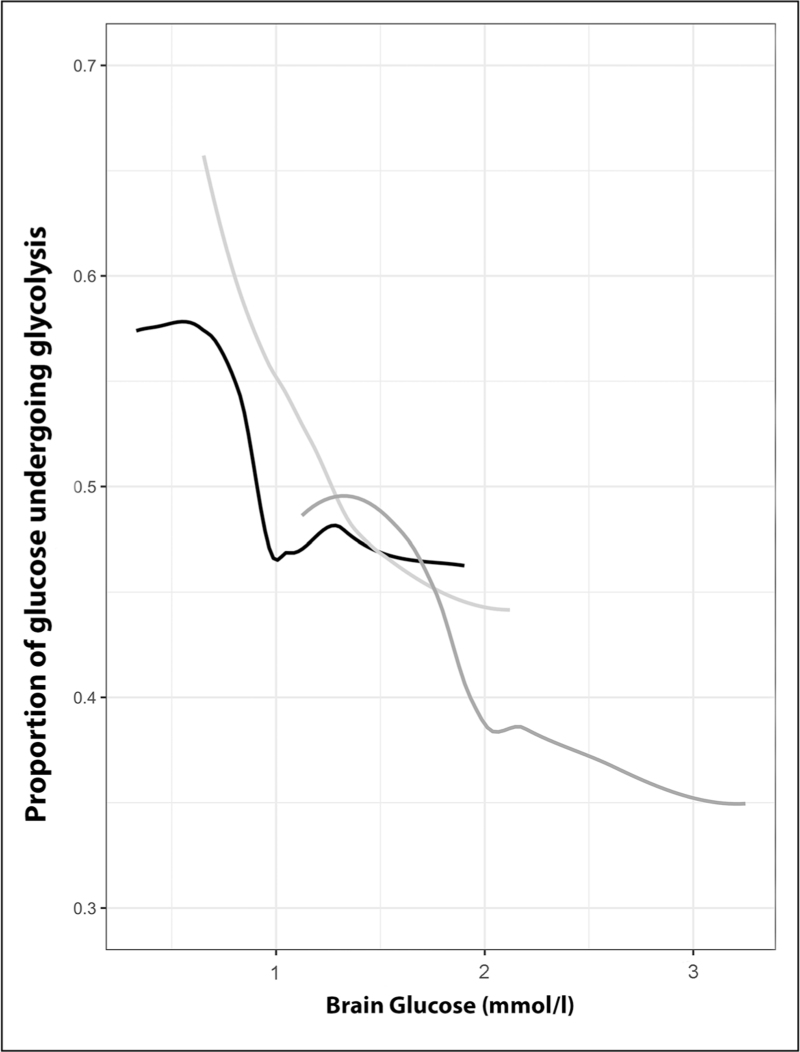
Impact of low glucose delivery on glycolysis. The relationship between the proportion of glucose that undergoes glycolysis and glucose within brain tissue modeled using locally weighted scatterplot smoothing. Data shown are within lesion core (black), penumbra (light gray) and peri-penumbra (dark gray) regions following traumatic brain injury.

Glucose utilization is dependent on delivery, and ^18^F-FDG PET data demonstrate that the proportion of glucose that undergoes metabolism increases as brain glucose falls below 1–2 mmol/l, particularly within lesions (Fig. 4) [5^▪▪^]. Other studies have shown increases in glucose metabolism with tight glycemic control, associated with an increase in the microdialysis LPR (>25) indicative of metabolic stress [27]. Randomized controlled trials of intensive insulin therapy in TBI show no benefit [28], and data from a recent PET study suggest that plasma glucose levels should not be allowed to fall below 6–8 mmol/l, ideally in combination with hourly bedside monitoring of brain glucose using microdialysis to ensure values of 1–2 mmol/l are maintained [5^▪▪^]. Such management is also supported by international consensus guidelines [16].

### Alternative energy substrates

Increased tissue lactate can be utilized as an energy substrate, particularly when glucose availability is low, and experimental and clinical TBI studies have shown improvements in CBF and cerebral metabolism following infusion of hypertonic sodium lactate that are unrelated to its osmotic effects [11]. Ketone bodies can provide an alternative energy substrate that bypasses glycolysis to be directly metabolized via the tricarboxylic acid cycle, leading to improvements in oxygen metabolism that support mitochondrial function, reducing oxidative stress and glutamate induced injury, and decrease the risk of posttraumatic seizures [29]. Studies on enteral ketone administration in TBI have shown safety and feasibility [30–32]. Other strategies to support mitochondrial function include substrates such as succinate that bypass complex 1 of the electron transport chain, which has been shown to be sensitive to damage following sepsis and TBI [33]. Experimental clinical TBI studies have used focally administered ^13^C-labeled disodium succinate to demonstrate succinate metabolism plus a reduction in microdialysis LPR and glucose sparing, suggestive of improvements in energy metabolism [34^▪^,^35^]. However, evidence from the cardiac and stroke literature demonstrate that the driver of reperfusion injury is succinate accumulation within mitochondria during ischemia. Following reperfusion, succinate is rapidly oxidized by succinate dehydrogenase (SDH) generating reactive oxygen species that result in injury and cell death [36]. Preservation of glycolysis in the face of mitochondrial dysfunction is protective during ischemia and reperfusion, and experimental data following TBI suggest that a reduction in mitochondrial electron flux and increase in glycolysis are neuroprotective. Succinate accumulation and oxidative injury can be ameliorated through inhibition of SDH, and the use of SDH inhibitors in an experimental model of TBI led to improved survival [37]. Given these conflicting findings it is difficult to recommend alternative energy substrates such as succinate following TBI without further research and evidence that confirms benefit.

## CONCLUSION

The combination of continuous bedside cerebral microdialysis and brain tissue oximetry with advanced neuroimaging has helped describe the spatial and temporal pattern of metabolic derangements, which are associated with poor outcome following TBI. While mitochondrial dysfunction and the use of alternative energy substrates are clearly potential targets for therapeutic intervention, improved understanding of the causes, impact and significance of metabolic derangements in clinical TBI are needed. The absence of therapeutic interventions that are proven to improve functional outcome means we should prioritize the maintenance of adequate oxygen and glucose delivery across the injured brain since this may accelerate the recovery of mitochondrial function and cerebral energy metabolism following TBI.

## Acknowledgements

*None*.

### Financial support and sponsorship

*The work was supported by the NIHR Cambridge Biomedical Research Centre (BRC-1215-20014). The views expressed are those of the authors and not necessarily those of the NIHR or the Department of Health and Social Care. J.P.C. was supported by grants from the UK Medical Research Council, the Royal College of Anaesthetists/British Journal of Anaesthesia, National Institute of Academic Anaesthesia and the Wellcome Trust. Cambridge University Hospitals NHS Foundation Trust and the University of Cambridge acted as the sponsor for research studies conducted by J.P.C., with responsibility for study conduct and management. Neither the funders nor the sponsor had input into study design; data collection, data analysis, data interpretation; writing of the report; or the decision to submit this work for publication.*

### Conflicts of interest

*There are no conflicts of interest*.

## REFERENCES AND RECOMMENDED READING

Papers of particular interest, published within the annual period of review, have been highlighted as:

▪ of special interest
▪▪ of outstanding interest
